# Squamous cell carcinoma arising from a cholesteatoma of the maxillary sinus: a case report^⋆^^⋆^

**DOI:** 10.1016/j.bjorl.2024.101408

**Published:** 2024-02-22

**Authors:** Tae-Gyun Kim, Chang-Ho Whangbo, Jae-Ho Yoo, Hee-Jun Park, Sang-Yen Geum, Seung-Heon Shin, Mi Kyung Ye

**Affiliations:** Catholic University of Daegu, School of Medicine, Department of Otorhinolaryngology-Head and Neck Surgery, Daegu, Republic of Korea

## Introduction

Cholesteatoma is a relatively common disease entity that usually occurs in the cavity of middle ear; however, it is rarely detected in the Paranasal Sinuses (PNSs). Cholesteatoma diagnosis solely based on clinical, or imaging findings is difficult; thus, confirmation is made histologically after the surgery. Sino-nasal Squamous Cell Carcinoma (SCC) can cause severe morbidity as it invades the surrounding structures, including the orbits and intracranial space. Although cholesteatoma and SCC involve the squamous epithelium, malignant change in cholesteatoma is very rare. We treated SCC in a patient with a cholesteatoma of the Maxillary Sinus (MS) who was not followed up after surgical treatment. To the best of our knowledge, this is the first reported case of an SCC arising from an MS cholesteatoma.

## Case report

**Fig. 1:**
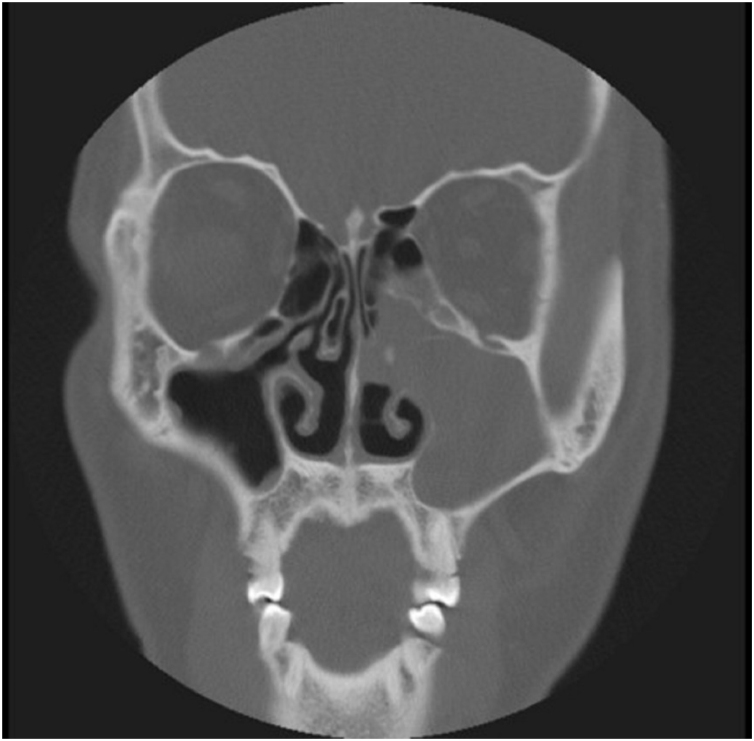
Radiology findings at the first visit, the paranasal sinus computed tomography coronal view revealed a nonhomogeneous soft tissue density in the left maxillary and ethmoid sinuses without bony remodeling.

**Fig. 2:**
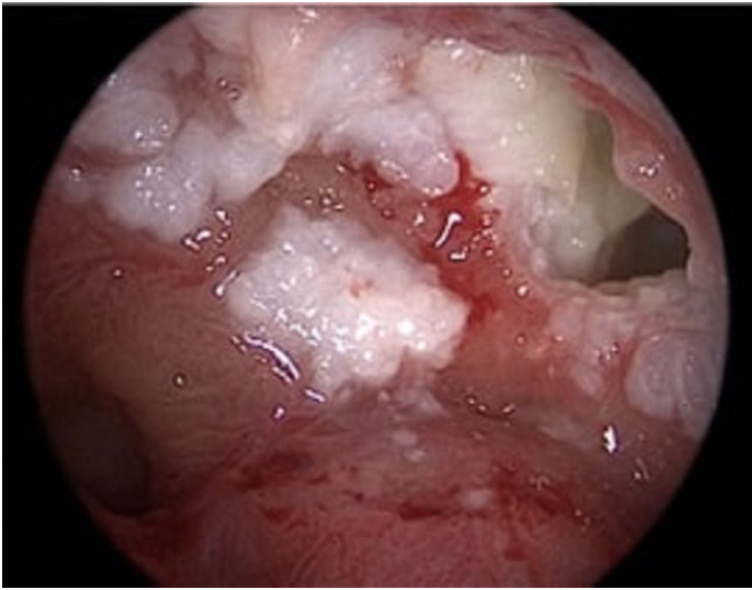
Nasal endoscopic findings 3-years after the endoscopic sinus surgery (1st surgery). Whitish keratinous material observed around the left maxillary sinus opening.

**Fig. 3:**
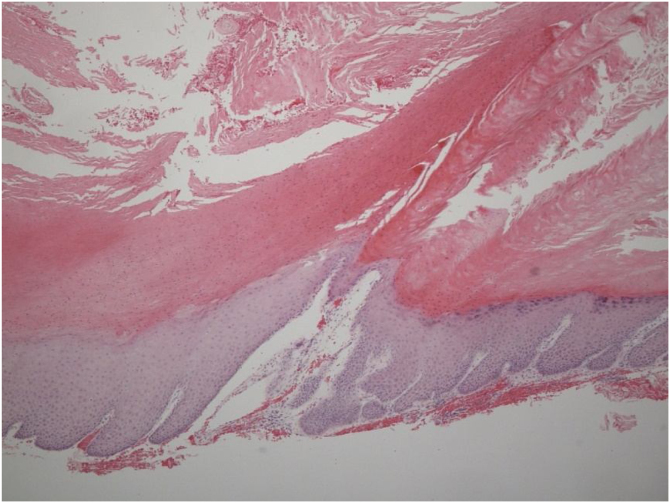
Histopathologic findings from Caldwell-Luc operation (2nd surgery). Microscopic examination revealed benign keratinized squamous lining with hyperkeratosis, parakeratosis (Hematoxylin and Eosin stain ×50).

**Fig. 4:**
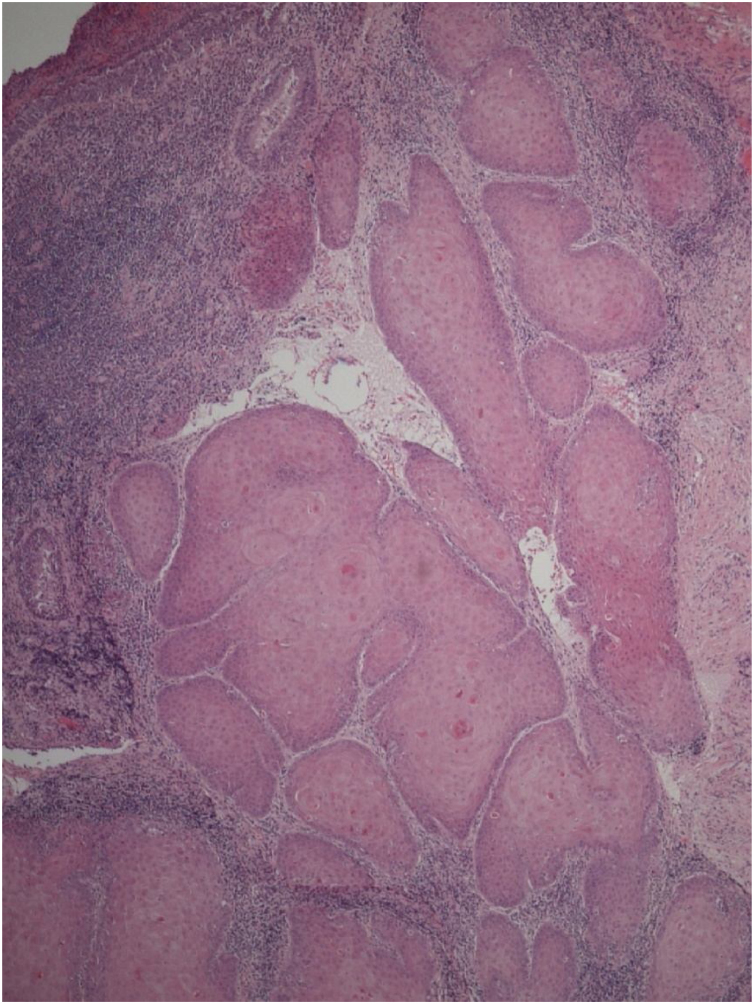
Histopathologic findings from the wide local excision (3rd surgery). Microscopic examination revealed irregularities in the basement membrane contour, along with the loss of cellular polarity, indicating well-differentiated squamous cell carcinoma (Hematoxylin and Eosin stain ×50).

**Fig. 5:**
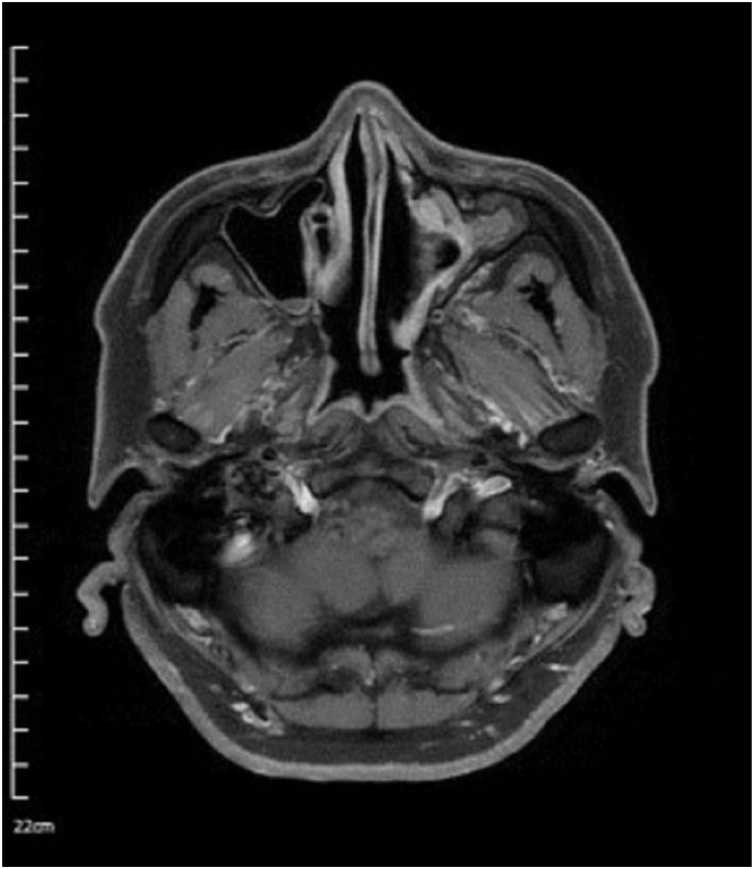
Radiology findings after the wide local excision (3rd surgery). The paranasal sinus magnetic resonance imaging axial T1-weight contrast-enhanced view revealed a thickened contrast-enhanced mass along the mucosa of the maxillary sinus and nasolacrimal duct.

A 40-year-old Asian man presented with repeated episodes of left sided dull orbital pain and a foul-smelling nasal discharge persisting for several years. He had often undergone treatment for sinusitis at other hospitals. There was no history of facial trauma and nasal or dental surgery. Nasal endoscopy revealed polyps in the left middle meatus and a purulent discharge. PNS Computed Tomography (CT) revealed a nonhomogeneous soft tissue density in the left MS without bony remodeling (Fig. 1). The histopathological examination of the specimen following Endoscopic Sinus Surgery (ESS) revealed keratinous material that was diagnosed as a cholesteatoma. The foul-smelling nasal discharge and intermittent facial pain recurred 8-months after ESS. Nasal endoscopy revealed white keratinous material around the natural ostium of the left MS. Caldwell-Luc procedure was performed 3-years later owing to persona circumstances of the patient. During the operation, the MS opening, and antrum were found to be filled with foul-smelling keratinous material (Fig. 2). The entire MS mucosa was removed, and heavy saline irrigation was performed. Histopathological examination revealed a benign squamous epithelium-lined lesion with keratin (Fig. 3). The postoperative period was uneventful; however, close follow-up after the surgery was not possible because of the circumstances of the patient, during a visit 4-years after the Caldwell-Luc procedure, nasal endoscopy revealed a potentially cancerous lesion on the lateral wall of the nasal cavity around the Nasolacrimal Duct (NLD). PNS CT revealed increased soft tissue density in the left MS and NLD, along with bony remodeling of the left MS due to the previous operation. A frozen biopsy collected from the lesion under general anesthesia confirmed cancer. Endoscopic wide excision of the medial MS wall was performed. Histopathologic findings revealed a well-differentiated SCC (Fig. 4). Postoperative cancer staging was performed. PNS magnetic resonance imaging revealed a thickened and contrast-enhanced mass along the mucosa of the MS and NLD; therefore, the possibility of residual cancer could not be ruled out (Fig. 5). Neck and chest CT with enhancement and positron emission tomography-CT showed absence of nodal or distant metastasis; therefore, the tumor was classified as stage II (T2N0M0). The multidisciplinary treatment team of the hospital, comprising specialists in otolaryngology, oncology, radiation-oncology, nuclear medicine, and radiology, decided to perform adjuvant chemoradiotherapy to prevent recurrence. This decision considered the circumstances of the patient who encountered difficulty in attending regular follow-up appointments and did not want the surgery to cause facial deformity. A combination of radiation (66Gray, 33-fractions) and chemotherapy (cisplatin, 5-fluorouracil) was performed. There was no recurrence 1-year after completing treatment.

## Discussion

Cholesteatomas are epidermal inclusion comprising desquamated debris with a keratinized squamous epithelium lining. There are 42 cases of PNS cholesteatoma in the English medical literature: 17 in the frontal sinus, 15 in the MS, 5 in the ethmoid sinus, 3 in the sphenoid sinus, and 2 in the concha bullosa.^1^ Four basic theories have been proposed to explain the development of cholesteatoma: congenital epithelial rests, squamous metaplasia, immigration, and implantation theories; however, none can describe the formation of PNS cholesteatoma. Previous reports have often associated PNS cholesteatomas with a history of surgery, trauma, or unerupted teeth, but our case was unique in that there were no such histories.^2^

Although cholesteatoma is benign, it can destroy bone and surrounding tissue; therefore, surgical removal is recommended to prevent recurrence, The surgical approach for MS cholesteatoma must be planned considering CT findings, which include size, location, and extent. In this case, cholesteatoma was not suspected in the first surgery, the MS mucosa was preserved and the natural ostium of the MS was widened by conventional ESS. After 3-years, Caldwell-Luc operation involving the removal of the entire sinus mucosa was performed. The patient was lost to follow-up and cancer was discovered 4-years after the second surgery.

Malignant change in a cholesteatoma is very rare. Six cases of temporal bone SCC associated with cholesteatoma have been reported in the literature. Several mechanisms underlying the progression of cholesteatoma to SCC have been proposed and most are believed to result from localized chronic inflammation, usually caused by bacterial infection such as *Pseudomonas aeruginosa*, or human papilloma-virus infection.^3^ However, the exact pathophysiology association remain unclear. To date, only five cases of SCC arising from a PNS cholesteatoma have been reported, all of them occurring in the frontal sinus.^4^ To the best of our knowledge, this is the first case of an SCC arising from an MS cholesteatoma. The prognostic factors of PNS SCC include disease stage, site or origin, histopathology and patient characteristics. Patients with early-stage disease or young age (<50) have shown better survival than those with advanced-stage disease or old age. The benefit of surgery and radiation was similar to that of surgery alone. Overall the 5-year and 10-year cumulative observed survival were 30.2% and 21% respectively.^5^

## Conclusion

Cholesteatoma is a common disease in the middle ear cavity, but it is rarely found in the PNSs. Although extremely rare, cholesteatoma can undergo malignant transformation. Awareness regarding the possibility of a cholesteatoma occurring in the PNS and its potential to become malignant can help reduce morbidity and mortality rates. We treated successfully a case of SCC arising from a cholesteatoma of the MS and report it with literature reviews.

## Funding

The author(s) received no financial support for the research, authorship, and/or publication of this article.

## Conflicts of interest

The authors declare no conflicts of interest.

## Meeting of ethical standards

This study was approved by the Institutional Review Board and Ethics Committee of Daegu Catholic University Medical Center (CR-23-135).
